# The Parenting Experience of Those With Borderline Personality Disorder Traits: Practitioner and Parent Perspectives

**DOI:** 10.3389/fpsyg.2020.01913

**Published:** 2020-08-07

**Authors:** Abigail Dunn, Sam Cartwright-Hatton, Helen Startup, Alexandra Papamichail

**Affiliations:** ^1^School of Psychology, University of Sussex, Brighton, United Kingdom; ^2^Sussex Partnership NHS Foundation Trust, Sussex Education Centre, Hove, United Kingdom; ^3^Department of Health Service and Population Research, Kings College London, London, United Kingdom

**Keywords:** Borderline Personality Disorder, mental health, parenting, practitioners, qualitative

## Abstract

**Background:**

Borderline Personality Disorder (BPD) is associated with challenges around emotional intensity and interpersonal difficulties. The children of parents with BPD are at risk of poorer outcomes in terms of their own mental health, educational outcomes and wellbeing. The challenges of being a parent can also exacerbate the symptoms of those with BPD traits. There is a pressing need to understand the experience of these parents and to determine what support would be appropriate and useful.

**Aim:**

To explore and compare the experiences and support needs of parents with BPD traits with the experiences and understanding of practitioners who work with them.

**Methods:**

Interviews with 12 parents with BPD traits and 21 practitioners with experience of working with individuals with BPD. The two strands of interviews were analyzed independently using a thematic framework approach, after which the superordinate and subordinate themes were subject to comparison.

**Results:**

Parents with BPD traits represent themselves as experiencing considerable challenges in their role as a parent. These included the impact of emotional intensity, social isolation and lack of a positive parenting models to draw upon. Practitioners demonstrated a strong degree of shared understanding into these difficulties. Both groups highlighted a lack of appropriate support for these parents.

**Conclusion:**

This research highlights the clinical need for parenting-focused support for individuals with BPD traits. Preliminary suggestions for format and content are given.

## Introduction

Borderline Personality Disorder (BPD) is a chronic and enduring presentation which is characterized by struggles with emotional intensity, fluctuation in moods, challenges in interpersonal relationships, heightened sensitivity to stress, and an increased likelihood of self-harm, substance abuse and suicide (Cheng et al., 1997; Skodol et al., 2002). The community prevalence of BPD is in the region of 0.5% (Coid et al., 2006) but individuals with BPD are disproportionately represented in both outpatient (Beckwith et al., 2014) and inpatient care (Zimmerman et al., 2005). The etiology of BPD is complex, and while there is some, though inconclusive, indication of genetic and biological factors (see Chanen and Kaess, 2012 for review), no single cause has been identified. However, there are a number of models which propose that BPD arises as the result of environmental risk factors on an underlying vulnerability (e.g., Linehan, 1993; Paris, 1994; Fonagy and Bateman, 2008; Crowell et al., 2009). In particular, BPD is associated with parental psychopathology and adverse childhood experiences, including trauma, abuse and neglect (Bradley et al., 2005). As such, when an individual with BPD becomes a parent, they do so in the context of their own frequently negative experiences of being parented and the likely lack of a “good” parenting model. When this is coupled with the challenges of ongoing life stresses, parents with BPD may struggle to know how to get alongside the needs of their children and, furthermore, most do not receive adequate support to do so (for review see Stepp et al., 2012).

A growing body of research into the parenting provided by individuals with BPD indicates patterns of behaviors that can hinder the parent-child relationship and place children at increased risk of negative outcomes (see Florange and Herpertz, 2019 for review). Parents with BPD can demonstrate impaired ability to recognize the emotions of their infants (Elliot et al., 2014) and are more likely to respond in an invalidating way to the ‘negative emotions’ of young children (Kiel et al., 2017). Parents of older children demonstrate, on average, lower levels of mind-mindedness and greater levels of overprotection and psychological control (Barnow et al., 2006; Schacht et al., 2013; Zalewski et al., 2014). These difficulties are reflected in poorer outcomes for children and adolescents in terms of behavior, affect, mental health and the parent-child relationship (Petfield et al., 2015; Eyden et al., 2016). Ultimately, children of parents with BPD are at greater risk of developing psychiatric symptoms, with parenting behaviors likely to be a contributing factor (Stepp et al., 2012; Eyden et al., 2016; Steele et al., 2019). For parents with BPD, impaired emotional availability and oscillation between hostile control and passive aloofness have been proposed as potential mechanisms for intergenerational transmission of poor mental health (Stepp et al., 2012; Florange and Herpertz, 2019).

At times, parenting is challenging for everyone, but parents with BPD report particularly high levels of parenting stress and low levels of competency, self-efficacy and reward in the role (Newman et al., 2007; Elliot et al., 2014; Ramsauer et al., 2016). Furthermore, there is a potential bidirectional relationship between family context and symptoms: Children of parents with BPD are more likely than controls to have disruptive behavior disorders and Attention Deficit Hyperactivity Disorder (ADHD), to have higher rates of BPD symptoms and greater levels of aggression and delinquency than children whose parents did not have a psychiatric disorder (Feldman et al., 1995; Weiss et al., 1996; Barnow et al., 2006; Huntley et al., 2017). Parenting a child with psychological, emotional and/or behavioral difficulties is stressful and has the potential to worsen a vulnerable parent’s own mental health (Berg-Nielsen et al., 2002).

While much of the research on parents with BPD has focused on individuals who meet diagnostic criteria for BPD, there is a case to extend the research parameters to incorporate those with subthreshold symptoms. The evidence suggests that individuals who fall below diagnostic threshold but still demonstrate some of the characteristics of BPD remain at risk of a range of negative psychosocial outcomes (Zimmerman et al., 2012; Kaess et al., 2017; Karukivi et al., 2017). In a series of studies, Zimmerman and colleagues have found that, in comparison with patients who meet none of the DSM-IV BPD criteria, a single BPD criterion is a significant predictor of a range of psychosocial morbidities (Ellison et al., 2015). This is echoed in the domain of parenting where the evidence suggests that parents with sub-threshold diagnoses and their children are likely to be at risk from some, if not all, of the parenting challenges seen in individuals with a full BPD diagnosis (Macfie, 2009). Studies that have included subthreshold BPD as well as full categorical diagnosis have found that maternal BPD symptoms that fall below diagnostic level are associated with psychological control of adolescent offspring (Zalewski et al., 2014; Mahan et al., 2018) and that sub-threshold BPD in parents significantly predicts BPD symptoms in young adults (Barnow and Arens, 2011). In terms of engaging with parenting needs, it is arguably these traits and behaviors, rather than diagnoses that should be the focus of a comprehensive risk assessment and with service provision (Adshead, 2015). As such, the present study focuses on individuals with both a diagnosis of BPD *and* those with subthreshold presentations. For clarity, this combined group will be referred to as parents with BPD traits.

Although the parenting challenges faced by individuals with BPD traits have been identified using a range of observational and self-report paradigms (see Florange and Herpertz, 2019 for review), far less attention has been given to recognizing how these parents understand and represent their own experiences, in particular using qualitative methodologies. For example, an extensive review of the qualitative research on mothers with severe mental health problems identified only one paper that included participants with BPD (Dolman et al., 2013). This gap is of particular relevance given that BPD is one of the most stigmatized mental health disorders: individuals who have BPD traits frequently report an uneasy dynamic with services, where their behaviors can be misinterpreted and misunderstood (Kealy and Ogrodniczuk, 2010; Black et al., 2011). In parenting support settings, this can be reflected with poor levels of engagement and high attrition in people with BPD: understanding their perspective is critical.

Two recent studies have employed a qualitative approach to explore the parenting experience of parents with BPD. Zalewski et al. (2015) and Bartsch et al. (2016) identified themes relating to low self-efficacy and satisfaction, disruption to empathic and emotionally validating responding, and difficulties in interpersonal boundaries. Both studies highlighted the lack of suitable parenting support for parents, with Zalewski exploring the acceptability of a DBT-focused parenting intervention with parents, and Bartsch generating a speculative set of recommendations from parents.

The current study extends this work by recruiting a broader community sample of parents, as opposed to those who were participating within a specific treatment modality (dialectical behavior therapy) and, unlike the papers above, does so within the United Kingdom.

Every parent exists within an interconnected system, which includes their child, their wider family and social network and any structural support they may receive. The current study seeks to gain qualitative understanding of the experience of parents who have BPD traits. It seeks to achieve this understanding alongside, and informed by, the experience of other factors within this system, namely practitioners who work with individuals with BPD traits.

While there is considerable research into practitioners’ general attitudes toward individuals with BPD (in which historically negative attitudes have shifted somewhat, e.g., Cleary et al., 2002; Treloar, 2009; Black et al., 2011; Day et al., 2018), only a handful of studies have explored clinician attitudes toward parents with BPD (e.g., Bartsch et al., 2015; Wilson et al., 2018). These studies drew upon survey data (Bartsch et al., 2015, 2016) or were drawn on small samples and focused solely within Child and Adolescent Mental Health Services (Wilson et al., 2018). The current study benefits from a substantially richer dataset generated through interviews and focus groups with a large sample of practitioners working in a range of settings.

The parent and practitioner datasets addressed the following research question: How do parents with BPD traits experience parenting and how does this compare with the way their experience is conceptualized by practitioners who work with them?

## Materials and Methods

### Ethics Statement

Ethical approval was obtained from the NRES Committee Brighton and Hove.

### Participants

#### Recruitment

##### Parents

Participants were recruited over a 6-month period (August 2018 – January 2019) from Sussex Partnership NHS Foundation Trust (SPFT) through the following mechanisms: self-referral in response to posters and flyers located in SPFT sites across Sussex; following promotion by clinicians; and via referral from another study seeking participants with some shared characteristics. To be eligible, an individual had to (i) be, or have been, a primary parental caregiver; (ii) be aged 18–89 years; (iii) have presence of traits associated with BPD (identified by a score of 4 or more on the BPD scale of the PDQ-4) a screening instrument for which a score of 5 is associated with a diagnostic level of BPD pathology); (iv) be under the care of SPFT; (v) be proficient in spoken English; and (vi) have capacity to provide informed consent to participate.

In total, 21 parents expressed interest in the study of which three chose not to continue to screening and two did not meet eligibility criteria on the grounds of scoring less than 4 on the PDQ-4-BPD (Hyler, 1994). Four eligible and consenting participants did not undertake interviews due to hospitalization, the effects of a change in treatment or disengagement (indicated by failure to respond to two phone calls and two emails).

##### Practitioners

Over a 6-month period (August 2018 – January 2019) practitioners working in mental health care, social care and the third sector were recruited through the following mechanisms: self-referral in response to promotional materials or following contact from the research team and snowball sampling (participants referring further participants from within their professional network). For a practitioner to be eligible they had to have direct experience of working with parents with traits associated with BPD.

Of 28 practitioners who expressed interest in participating, 21 went on to be interviewed or participate in a focus group. Of those who failed to participate: three were Social Workers within Children’s services; two were Child and Adolescent Mental Health Services (CAMHS) practitioners; and two worked within Adult Mental Health. These practitioners either stated they could not give up the time, failed to attend interviews/focus groups and/or disengaged.

#### Participant Characteristics

##### Parents

In total, 12 parents aged between 39 and 58 (*M* = 47.33, *SD* = 5.78) were interviewed of which 11 identified as White British and one as White Other. Parents scores on the PDQ-4-BPD ranged from 4 to 8 (*M* = 6.42, *SD* = 1.24). Two parents were male and ten were female. Parents had between one and five children (*M* = 2.17, *SD* = 1.11) and the age of children ranged between one and 34 years (*M* = 19.85 *SD* = 9.40) with seven participants providing care for dependent children (<18 years). Half of the group were married, in a long-term relationship or co-habiting, and the remaining six characterized themselves as single either following divorce or the death of a partner.

Education and employment varied across the sample with one parent stating they had left school before aged 14, five had attained GCSE or equivalent (school age 16 years), one had attained A-level of equivalent (18 years) and three had completed a first degree. At the time of interview four participants were in employment (part-time or full/time) and eight were unemployed.

##### Practitioners

Over half of practitioners were adult mental health practitioners (*n* = 13); six were in council-funded roles; one was employed by a charity and one by CAMHS. A range of roles were represented with six Occupational Therapists; five Social Workers; three Clinical Psychologists; three Nursing Professionals; two Family Coaches; one Midwife and one Charity worker. All of the practitioners had been working in their discipline for a minimum of 6 years, with 38 years the longest reported service duration (*M* = 18.89, *SD* = 9.09). Six participants identified themselves as having managerial responsibility.

#### Procedure

Following provision of informed consent, participants were either interviewed alone, in a pair, or as part of a focus group. The format was determined by participant choice and scheduling practicalities.

Ten parents were interviewed alone and two were interviewed as a pair. Interviews were conducted in the home (*n* = 4), or on NHS sites. Thirteen practitioners participated in team-based focus groups which ranged in size from three to six participants. Four participants were interviewed in multi-disciplinary pairings (two separate interviews) and four practitioners were interviewed alone. All except one interview took place on an NHS site.

In all cases the participants were interviewed by the lead researcher using a semi-structured topic guide developed in consultation with a clinician with expertise in parent-based work and a clinician with core expertise in supporting individuals with personality disorder (see Supplementary Material for topic guides). The topic guide was used as a framework to determine the overall interview content, but questions were developed dynamically in response to the answers and comments of participants. This approach was used to maximize the development of a relationship between interviewer and participant(s) and has precedence in health and psychological research (e.g., Brazier et al., 2014). Within the paired interviews, each question would be repeated to both participants, though space was available for them to comment upon and add to the answers of their co-interviewee. In the focus groups, questions were responded to directly by individual participants and/or formed the basis of a discussion within the group.

The parent topic guide was structured around three research questions: how do individuals with challenges around emotional intensity experience being a parent; what support have they sought and experienced and how effective and appropriate has it been; and what support would they like to receive/have liked to have received?

The practitioner topic guide was structured around the following research questions: how do practitioners conceptualize the parenting experience of individuals with BPD traits; what mechanisms and opportunities to support these parents are identified by practitioners; how do they experience working with parents with BPD traits?

Given the emotive nature of the subject, the interviewer maintained an empathetic and reflective stance. Interviews took between 45 and 65 min and were recorded on an encrypted Dictaphone. After each interview, participants were given the opportunity to raise any concerns or discuss any negative feelings the interview had raised.

Participants (not practitioners) were provided with a £10 voucher to thank them for their contribution.

#### Analysis

The data were anonymized and transcribed. Both sets of data (parent and practitioner) were, then subject to a framework analysis (Ritchie and Lewis, 2003). This form of thematic analysis has been used widely in social sciences research and is become more common in psychologically-orientated research. Framework analysis includes a structured and transparent data management and synthesis process. Utilizing this approach facilitates analysis across themes and across cases.

The framework approach comprises six stages: (1) familiarization with the data, (2) review of the dataset to identify recurrent themes or ideas, (3) development of a hierarchical thematic framework, (4) indexing – labeling/tagging the data to the framework, (5) organizing the data according to a revised version of the index to create a set of thematic matrixes, (6) summarizing the data using appropriate synthesis, which is applied to the whole dataset. This process is iterative and flexible (for worked example see Parkinson et al., 2016).

The lead researcher (AD) and a second researcher (AP) separately reviewed the data at each stage of the process and these perspectives were integrated iteratively into the thematic framework and the index. Additional oversight and comment were provided by the lead researcher’s supervisory team: who combined clinical and research experience with parents and personality pathology. The research team operated within a clinical-academic framework and the clinical and academic perspectives and identities of members inevitably shaped the methodology adopted and the interpretation of data. However, the research team adopted a position of epistemological reflexivity in which the team would engage with and question their methodological decisions. The study lead used a reflexive journal and reflexive matters were discussed within the team including direct engagement with assumptions and biases. For example, noting and exploring an occasion when a member responded negatively to a parent’s representation.

The matrixed dataset (stage 6) was then subject to a thematic analysis whereby the charted data was explored with the aim of identifying patterns which reflected the shared experiences of each group. This set of themes was discussed with the research team and revised. Parents were invited to meet with the research team to discuss the emergent themes. Four participants met as a group with the CI and provided feedback on the themes identified in the data. This took the form of the CI reading descriptions of the themes and example quotes and asking for parent’s reflections which were then used to further refine and name the themes. Participants were provided with a ϵ10 gift voucher as acknowledgment of their time.

Once analysis of each of the three data strands was completed, the superordinate and subordinate themes from the parents and practitioner datasets were compared (see Tables 1, 2). The results from these two sets of data have been integrated in the results.

**TABLE 1:**
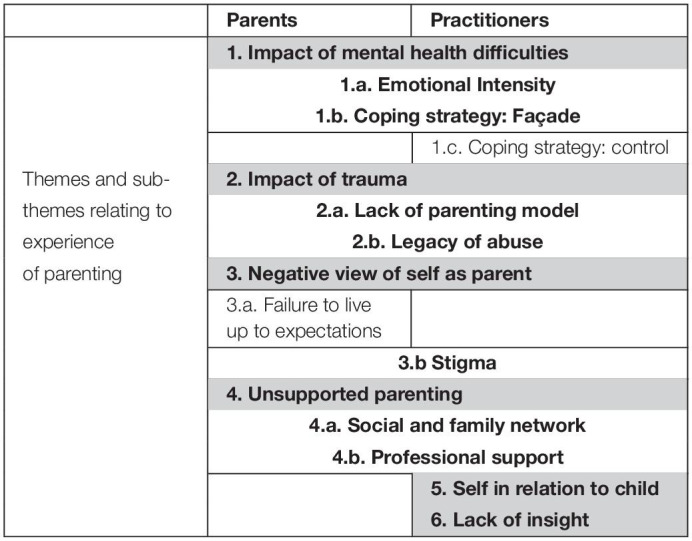
Grid of superordinate [shaded] and subordinate themes indicating which were shared by parents and practitioners [in bold] and those which are present only in the data from parents or practitioners [not in bold].

**TABLE 2:**
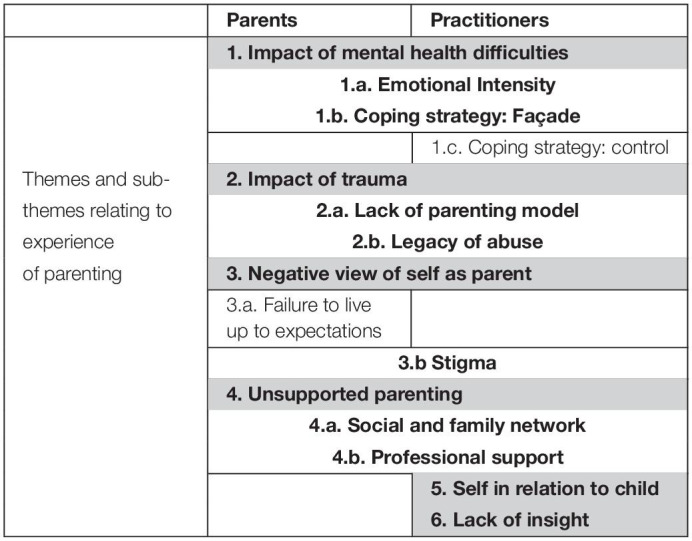
Grid of superordinate [shaded] and subordinate themes relating to parenting support needs, indicating those shared by parents and practitioners [in bold] and those which are present only in the data from parents or practitioners [not in bold].

Due to the sensitive nature of the accounts given, participants are identified only in broad terms: by family role or profession.

## Results

Exploring the parenting of individuals with BPD traits from the perspective of the parents themselves and practitioners who work with them revealed considerable shared understanding. Amongst parents, despite situational diversity, there were clear commonalities in the way they made sense of their experience of parenting. These were frequently echoed in the descriptions of practitioners. The main themes identified in both sets of data are orientated to challenges in the parenting role. Comparison of the data revealed four shared superordinate themes with two additional themes present only within the practitioner interviews (see Table 1).

**1. Impact of mental health difficulties**

In the accounts of both parents and practitioners, parents were characterized as struggling to manage the impact of their mental health difficulties on their ability to parent in the way they wished. For both parents and practitioners this was present in the way that parents related emotionally to their children and their children’s emotions. A second subordinate theme was the deployment of a facade as a coping strategy. For practitioners, an additional coping-related theme was over-planning and control.

**1.a. Emotional intensity**

For parents, the struggle to experience and contain their own emotions directly related to their ability to respond to their children and their children’s emotions, particularly when their children were distressed. Strong and uncontainable emotional responses, usually anger, despair or emotional withdrawal could be generated by things their children said or did: for example, one mother described her response when her daughter told her that she hated her:

“*I would cry and cry and, you know, just think about it constantly for weeks.*”

Within practitioner accounts, parents’ difficulties in managing their emotions and responding to the emotions of their children was depicted as a core characteristic of parenting, as one Midwife stated:

“*It’s that impulsivity, isn’t it? And that quick, that sort of, that quick escalation of their emotional intensity that is usually triggered by maybe something that their child might’ve done and then the way that they misinterpret it and that causes the anger and frustration and the way that they respond to the child and it may not always be helpful for the child*.”

This describes the complex interplay of emotional misinterpretation and responsiveness present in the accounts of both parents and practitioners. This was depicted as particularly heightened when parents responded to the emotions of their children; parents described responding with intense anger and distress, and by minimizing and/or rejecting their children’s emotions. As one mother described: “*I couldn’t cope with their emotions and I couldn’t cope with mine either*.” In disclosing these responses parents frequently expressed dismay:

“*Then suddenly I find it makes me really irritable, and angry and then I am getting cross with her and I am making it worse and then it actually feels, oh my god this actually this feels quite abusive, I am shouting and being mean to a child who is here actually having a panic attack*.” (Mother)

Practitioners also described this pattern of uncontrolled response followed by regret:

“*Because of their emotional intensity, it’s almost if they say: ‘Well I can’t help myself, I end up shouting at them.’ And you know, then, the guilt again kicks back in*.” (Occupational Therapist)

Specific developmental transitions were identified by both parents and practitioners as being associated with emotional dysregulation. While the transition to toddlerhood and school were mentioned, adolescence was most frequently identified as a period in which parents struggled to manage their emotions. This was specifically associated with feelings of rejection caused by their child’s need for increased independence. This was reflected in the accounts of practitioners as described by a Family Coach:

“*As the kids get older, parents have real difficulties with managing that. You know, there is a potential for flare ups, for big arguments, for all kinds of stuff. For violence, for abusive behaviours. You know, the resumption of alcohol or substance abuse to manage the feelings they are having, how bad the relationship is, the disappointment they can feel*.”

**1.b. Coping strategy: façade**

Parents described putting on a façade to manage the challenging interplay of parenting and mental health. The need to present an alternative or masked version of the self was directly related to their belief that “*there was something wrong*” (Mother) with them, that they were different to other parents who just “*sailed through things*” (Mother). These differences exposed them to stigma or the threat of child removal. The presentation of a more readily understood and acceptable version of themselves was perceived as protective and necessary in accounts of their engagement with the outside world, as one Mother described:

“*I would be planning [suicide] and, you know, things I would be doing and on the flip side thinking about who or where I was going to with regards to Rainbows and Pottery Club. You know, it is two different worlds for me*.”

This parenting façade was depicted as protective but also exhausting and participants were uncertain about its effectiveness in convincing the outside world or their children:

“*She [child] would say sometimes, she would say: ‘Why can’t you be happy like you are when you are at school?’*” (Mother)

Practitioners also identified the maintenance of a façade, “*a sort of masking*,” as a common but unsustainable coping strategy developed in response to fear of judgment:

“*I felt so much for some of the mothers that I’ve worked with…when you can see why they’re doing it, the child looks impeccable, you know? And you know why, you know, the bow in the hair, you know, the absolute, you know, beautiful clothes and you just think. Gosh, the pressure that they must feel under to do that*.” (Social Worker)

The use of a façade as a method of managing fear was seen by practitioners as ultimately detrimental as it prevents parents from opening up about their need for support or practitioners recognizing a need to offer it. As one Occupational Therapist described, this led them to work as a “*detective*” to unpick what is “*actually going on at home*.”

**1.c. Coping strategy: control**

In describing the parenting provided by individuals with BPD traits, practitioners identified control as a coping mechanism. Parents were characterized as deploying strategies such as over-planning to enable them to manage their own heightened responses to a situation – this could relate to the organization of time and activities and rigidity in daily routines, as a charity worker described:

“*They can over plan, over trying to keep safe so they can get completely thrown when that doesn’t happen or something else comes in. So, it is almost like I am OK if I plan out my week or my day but then the crisis.*”

The effort to control was seen as most effective when children were young; as a Family Coach described, “young child can be fairly easy because you are in a position of power.” As with maintaining a façade, practitioners felt the level of control exerted was largely unsustainable. The failure of control was associated with disengagement and the decision “*not to do any kind of job at all*” (Family Coach).

**2. Impact of trauma**

Many of the parents described childhoods lacking nurture and love or characterized by anger and violence. Some experienced abuse in childhood or adolescence, often perpetrated by individuals within their family. For parents, their experience of being a parent was directly related to the maladaptive parenting they had experienced, to traumatic early life experiences, or both. This generated two subordinate themes: lack of an appropriate and nurturing model of parenting to draw on and the aftereffects of abuse. The legacy of these two overlapping themes is viscerally present in the recollection of one mother: “*I didn’t know how to love them but I didn’t want anyone to hurt them.*”

Practitioners also represented parents as shaped by the lack of nurture and/or trauma they had experienced as children. As one described: “*I can say most of my patients, most, maybe all of them have had horrific, awful lives. And then they just try and struggle through*.” (Community Psychiatric Nurse)

**2.a. Lack of parenting model**

Most parents explicitly described the absence of a positive parenting model to draw upon. In some cases parents tied this to their own inability to parent, for example not being able to play. A number described consciously attempting to provide a better form of parenting to their children, as one mother described:

“*Because you always think you are going to, to do better. You kind of think you can always overcome it. Anything. And be the person you want to be for your children and do better than your parents did*.”

However, for some parents, trying to do things differently meant doing the opposite of what they had experienced:

“*Yeah, I think it’s, you know, my mom was one end of the scale and I was at the other end, I think. There should have been some kind of middle.*” (Mother)

Practitioners also described parents who often lacked a positive model of parenting to draw upon and the detrimental effect that had on the care they could provide to their children.

“*And what do we do about the fact that our clients themselves have problems being, had poor parenting themselves and, you know, so they’re almost sort of like passing on what they know because that’s all they’ve know*.” (Occupational Therapist)

However, practitioners also recognized that many parents consciously attempted to do things differently, to offer their children an improvement on their own experience. Though these efforts again could be compromised:

“*If somebody’s not had good parenting experiences and then in their desperation to try and get it right, they get completely off kilter*.” (Senior Occupational Therapist)

At the core of practitioners’ accounts of the impact of the lack of a parenting model was the risk to the next generation. As a Midwife described:

“*We literally do have mum and daughter [in our service], don’t we sometimes? Yeah. And soon, it will be their grandchildren and granny*.”

**2.b. Legacy of abuse**

The second subordinate theme relates to the impact of abuse. Parents who had experienced abuse represented it as having a central effect on the parenting they provided for their children. The impact of early abuse was depicted as having a complex legacy as parents struggled to manage their own responses to their past. For example, as one mother described, this could be heightened when a child reached the age at which their own abuse started:

“*When my daughter turned four, I was, was sexually abused and it started at the age of four, and it messed me up a lot. And I would look and think how could somebody do something like that? And then I lost the plot a bit and I was sectioned*.”

In one form or another, each of the parents stated: “*I don’t want any other child to experience what I have experienced*,” (Mother) which in some cases led parents to “*overcompensating*” (Father). In particular, this could take the form of overprotective behaviors. “*I didn’t trust them going out – even when they were 14*,” (Mother). For two parents, the legacy of abuse was identified in their struggle to provide physical affection:

“*When they reached certain age I found it very hard to cuddle them. I think it was about from six to seven up. Yeah, I was very tactile until that point.*” (Mother)

Practitioners also highlighted the burden of past trauma for parents that was related to being stuck at a developmental stage which made it hard to provide appropriate care:

“*Obviously it can come out in different ways for different reasons, but that is the most complex when there is something that has perhaps kept them somewhere in their development as a child so they are fighting to be a parent and a child*.” (Charity Worker).

More commonly, a legacy of abuse was that parents were “*desperately trying to do differently*” (Social Worker) and prevent repetition of what had happened to them:

“*’Cause like you were saying about when trauma started you have, uhm, a mother was raped at 15 and now her daughter is 15, so she’s now becoming extremely protective of the child and, you know, and not allowing them to grow up because they’re so frightened about what was gonna happen*.” (Occupational Therapist)

Practitioners also described an ongoing process of re-traumatization in which parents who had adverse experiences in their own childhood continued to experience a pattern of trauma and loss, for example from abusive relationships and, most saliently, in becoming a parent.

“*We get parents who the…kind of the process of becoming a parent is traumatic in many ways and so they’re dealing with their own trauma whilst also trying to put in the place the skills of containing it…a baby*.” (Senior Social Worker)

For practitioners the distressing, though not uncommon, conclusion to this pattern was the trauma of child removal.

**3. Negative view of self as parent**

Parenting was largely represented by parents as difficult and described in negative terms. This was often allied to a belief that they were not doing/had not done a good job, especially in comparison to others. Even for parents who went on to express some satisfaction in being a parent, their initial description of their experience was frequently negative, e.g., “*horrendous*” (Mother) “*a nightmare*” (Mother). When parents expressed pleasure in parenting, or some component of it, it was largely orientated around feelings of competence and teaching, as one mother described:

“*There are times when I find it fantastic and I feel like I am doing quite a good job and I am actually doing good by them, you know. Sometimes I might be taken to think I am giving them a better chance than others by helping certain things or helping them understand the world in a certain way, but there are other times when I can’t be the person I wish to be*.”

Consistent in this theme was the expression of sentiments relating to their failure to meet their own expectations, not being the “*person I wish to be.*” For many parents this was embedded in a reflective narrative in which their failures had led to negative outcomes for their children:

“*Certain things stand out in him now, which I can see I was like – in how I was a mum and how that has affected him really, to me in a bad way*.” (Mother)

This was mirrored in the accounts of practitioners who described parents struggling to cope with knowledge of the negative impact they had on the lives of their children, particularly when children developed mental health difficulties of their own:

“*You know, I saw a lady today and she just was like ‘I just want to write letters to everybody in my family.’ Because she has adult children now, but now she’s thinking: ‘Well she’s really struggling and I know that I’m, you know, I ran off, and I took overdoses, and I wasn’t there as a mum, and I did this, and I did that, and now she’s like this’*.” (Midwife)

For practitioners, the negative views parents held of themselves had been shaped through their formative experiences and subsequently reinforced. One charity worker described the way a parent’s experience of being passed around mental health teams reinforced her poor self-esteem:

“*The ‘end of the pile’ was explicitly used in a conversation I had with someone. Like a dumping ground – like now we are dumped in that pile*.”

The intensity of the emotions related to these feelings of failure coupled with the low self-esteem and guilt can for some parents become enmeshed in patterns of self-harm and suicidality.

“*Some things…drugs…either prescribed drugs or illicit drugs…alcohol. Self-harm, sometimes, in the moment can be a way of stopping intense emotions…can be a way of validating a sense of their own badness. A punishment, I’m not good enough*.” (Clinical Psychologist)

**4. Unsupported parenting**

Most participants characterized themselves as isolated and lacking support from a personal network and this was depicted as either of their own choosing, a consequence of their mental health, or both. Most parents described having few if any friends and most had experienced relationship-breakdown. Two parents identified supportive relationships with their co-parent which were informed by the co-parent’s understanding of the participant’s difficulties. One participant derived support from their child who had taken on caring responsibilities from a young age. However, only one participant was able to recount a positive experience of parenting-focused support, which was via a charitable organization.

Typically, parents represented mental health services as unengaged with their needs as a parent. If parents did share a need for assistance, it was unavailable. More than one parent described asking for help, to be told that there was not any. Another described a perception amongst health professionals that parenting is something that: “*you just get bloody used to*.” (Mother)

Difficulties in navigating the system, understanding the pathways to support – or even in having knowledge that it existed – was also present across the interviews. For example, one mother stated she “…*didn’t know where you could look for support or if there was any support there for you*.” (Mother). For the male participants, engagement with support was further compromised by their gender, which they characterized as a barrier to access. If support was available, it was not accessible to them as fathers, in particular because they did not have the vocabulary to ask for it:

“*Unless you use the right words then help isn’t there, but they don’t tell you what the right word is, you have to wait and find out….if you use the word “I need help,” you’ll get help! But being a man you don’t think you need to use that word you just say “I don’t know what do about this*.” (Father)

In addition, participants expressed ambivalence about support, embodying a tension between desire to engage and fear of accessing support or asking for help. This was often rooted in a fear of child-removal. Two participants had experienced temporary child removal and for eight of the remaining ten parents, the risk and fear of child removal was extremely powerful, as a Mother described:

“*No. I never got no support. I was anorexic as well when I was pregnant. Errm. I was anorexic whn they were growing up in school. But nobody noticed anything. And I couldn’t ask for help. I can’t now. Because they might take the kids away.*”

An additional component of this ambivalent relationship with support related to parental expectation that services or groups would not be appropriate or would not understand then. This was highlighted by practitioners:

“*And I know that people feel so self-conscious, that those baby groups and those parenting groups aren’t for them. And sometimes they are actually right because they are going to feel very different and isolated if they go into some, you know some lovely group in Hove where it is all organic this and you know*.” (Family Coach)

Practitioners characterized parents as frequently isolated, lacking familial support and facing difficulties in accessing and of engaging with support. One practitioner described how these elements interact:

“*The thing that I have been thinking about is about how actually these guys hold their family systems together and the kind of reality that some of them might be struggling to hold the relationship down or just be single parents. And then that’s even harder logistically and to access treatment. Practically, to be able to do any of this stuff*.” (Clinical Psychologist)

In the accounts of practitioners, parents who do not have a positive network to draw upon are less likely and able to engage with support either for their mental health or their parenting. The absence of support can lead parents to depend on individuals who are harmful, which can take the form of dependence on parents who themselves are abusive, or in terms of romantic partners.

A charity worker also identified a pattern of dependence on mental health crisis support which could have been reduced by supporting parents in managing their family relationships: “*it is not really a mental health problem, it is a childcare issue which is not being managed.*”

**5. Self in relation to child**

Practitioners described parents as struggling to maintain a stable role in relation to their child. This was manifest in an enmeshment within the parent-child dyads as a Charity Worker stated.

“*So, it is a very intense relationship. And so they are trying to support or protect each other and you can see it is not always healthy but it is not an explosive or uhmmm it is just not healthy*.”

These interpersonal difficulties existed in the form of role reversal. An Occupational Therapist gave this example of a parent demanding care from the child:

“*So for me there can be this: ‘I’m your parent but you also have to look after me.’ So, it is a little bit more expecting a child to be good to them or to be looked after, you know, poor mummy has had a bad time*.”

For practitioners, role reversal could incorporate the child taking on a carer role. It was also represented as a method of behavior management and a means of eliciting affection. In some cases, the parent was described as being unable to fulfill their adult role to the child as a result of the legacy of their own maltreatment in childhood.

“*She was talking to her child, that she sounded just like a child herself, talking to her child. Like, kind of whiny voice like ‘I’m on the phone,’ you know, kind of child-like voice and I just thought ‘oh, is she talking to her parents?’ and then I realize, no, she’s actually talking to her daughter, young daughter*.” (Occupational Therapist)

**6. Difficulties in self-insight**

Practitioners frequently referred to limitations in parents’ insight into their behavior and the effect it may have on their children.

“*Externally looking outwards for some kind of, uhm, solution to the problems and find it really difficult to kind of…come back to themselves and identify a part in the chaos or what’s happening around them. And it’s this kind of desperate seeking often…and being caught up in what everybody else is doing. Sometimes, even the child themselves rather than where their part in it is*.” (Social Worker)

For parents who are involved with social care, being unable to understand their role in their family’s situation makes it difficult to change and increases the risk of child removal as a Social Worker described:

“*I think if we got into that dynamic where we can see really concerning things develop, uhm, it tends to be the situations where the parent’s ability to be mindful of that and having insight into that is quite impaired.”* (Senior Social Worker)

In the characterization by practitioners, parents may also lack insight into their child’s experience and understanding. Parents were described as sometimes struggling to “*put themselves into their children’s shoes.*”

Though insight was generally depicted as compromised in parents, it was highlighted that some parents had the capacity to develop a better understanding of themselves or their situation, in particular following therapeutic intervention. However, this new insight could be compromised by stress and emotional dysregulation.

### Themes Related to Support

Across both datasets, a mismatch is evident between the reported parenting experience and the parenting support available. Both parents and practitioners described a need for specific parenting/family related support, for this group of parents. In describing what would be beneficial and appropriate there was considerable overlap. However, parents and practitioners also identified separate areas where they believed support would be useful.

**1. Connection through shared understanding**

Both parents and practitioners articulated a need for support which was characterized by an understanding of parent experiences and challenges in terms of their mental health. Most parents wanted an opportunity to interact with other parents who had shared experiences of mental health. In some cases, this was related to their positive experience of participating in specialized group-based treatment (e.g., STEPPS-EI; STEPPS).

For others, the focus was on space where they would feel free to be open about the difficulties they faced, as one mother explained: “*If you’re with a group that you did not originally know but who understand, you could open up more*.” The two fathers also presented a desire to connect with other men with shared experience, in part because they had felt excluded in groups that were primarily female.

Practitioners, similarly, identified a need for parents to be able to share their experiences without fear of judgment:

“*Even offering them the space just to be in a, uh, group of parents…That space where they can just be parents and be okay for them to talk openly and honestly*.” (Occupational Therapist)

This was associated with ideas with shared learning and supporting parents to build a network: breaking down the perceived isolation of parents through “*a sense of belonging and identification with people*.” (Mental Health Nurse)

A subordinate theme, which was only present in the parent data, was the need for support to be facilitated by practitioners with an understanding of mental health and abuse.

**2. Accessible not just available**

For support to be effective it should be designed and implemented with an understanding of barriers to access, which include parents’ complex relationships with support as well as logistical concerns.

Parents identified cost, location, timing and availability of childcare as playing an important role in the impeding and facilitating engagement with support:

“*The financial aspect even though they were offering things on a financial scale, it was a barrier to be honest. And the fact that location-wise it meant long travel, which sounds really pathetic but when you are in the throes of a really intense lifestyle.”* (Mother)

Practitioners echoed this, describing the need respond to the specific logistical needs of parents, particularly those who may not have other support around them:

“*Then there comes the issue with we want parents to attend group work at our group therapy program but there’s never any childcare, or school holidays, or whatever. I mean, that again is a basic*.” (Social worker)

Alongside these practical considerations, a number of practitioners identified a need for a flexible approach, to take into account the complexity many parents were living with:

“*You have a boundary, definitely have a boundary, but have flexibility with the boundaries. It is not that you break it, but be flexible. But once you have got things like a bunch of restrictions, you know, agendas of whatever your service is. That rigidity, people with emotional intensity don’t do well with that. They don’t get it. They will struggle to understand it*.” (Mental Health Nurse)

**3. Support for children**

Parents identified a need for support to be targeted at their children, to enable the children to cope with their experiences. This included supporting children in their understanding of mental health and providing opportunities to connect with children with shared experiences:

“*For the children to be heard and to have other experiences with other children that had difficulties, so they had group support*.” (Mother)

Few practitioners identified children as a target of support. More commonly, children were described as at a distance from their work. Where support for children was discussed it was largely through the prism of the parent, either in terms of support for the parent having a cascading benefit to the wider family, or as a method of engagement:

“*I think an incentive is related to the guilt that some parents feel that their children are not meeting other children so giving a way to connect their children. That might be a nice thing*.” (Family Coach)

**4. Managing emotions**

Practitioners identified a need for support in terms of parents’ emotional regulation and in responding to the emotional needs of the children:

“*I feel on one hand they would need a way to regulate their emotions, maybe like, uh, mindfulness course. Something that really, like, or skills course that really calms down the nervous system*.” (Occupational Therapist)

While parents expressed difficulties in managing and responding to emotion, it was not a strongly identified factor in their desired support.

**5. Normalizing parenting**

Practitioners identified a need to encourage parents’ understanding of typical parenting experiences – that parents and practitioners at times pathologize parenting challenges which may be common and shared experiences:

“*Just normalise some of these intense reactions. Parents having intense reactions – hold the front page, kind of thing. And so, if some kind of distress is talked about in the context of their parenting it does normalise it a bit. Because every parent can go through it*.” (CAMHS, Clinical Psychologist)

## Discussion

This study aimed to generate a deeper understanding of the parenting experiences of individuals with BPD traits, as represented by parents and by practitioners with experience of working with them. Comparison of the experiences and views of these two groups revealed considerable shared understanding. Both parents and practitioners described in stark terms the challenges that parents face in managing their mental health while seeking to provide care to their children. While both groups identified a deficit in and barriers to appropriate support, there were also some clearly identified targets for engagement.

Parents and practitioners described the impact of mental health difficulties on parents’ ability to cope with the day-to-day demands and responsibilities of parenting. Within this theme, challenges around emotional intensity were frequently cited as causing a burden on families. The focus on emotional intensity as a primary characteristic of disordered parenting mirrors the views of researchers who implicate emotional dysregulation in the development and maintenance of BPD (Linehan, 1993; Glenn and Klonsky, 2009; Stepp et al., 2012). The association between emotional dysregulation and problematic behaviors such as aggression, binge-eating (Selby and Joiner, 2013) may be reflected in the presence of maladaptive parenting behaviors such as verbal aggression or withdrawing in response to emotionally intense familial situations. For parents, the recognition that these experiences were not “like other parents” and their awareness of the effect their mental health may have on their children, played into a cycle of guilt and despair. This, in turn, contributed to the negative opinion parents held about themselves, as well as exacerbating mental health difficulties. This pattern may also incorporate models of self-stigma whereby individuals with mental health difficulties internalize the negative labels associated with their disorder (for an example of relationships between public and self-stigma see Vogel et al., 2013).

A parent is part of a chain which links their past experiences of being parented to the care they provide to their own children. The majority of parents in the study lacked positive experiences of parenting to draw upon and had a history of trauma. Both parents and practitioners described formative experiences of neglect, abuse, lack of nurture or invalidation. These were clearly identified by parents and practitioners as playing a role in clients’ parenting. This absence of a positive parenting role model, and subsequent difficulties with emotional validation and over-protection, were also identified as areas of particular challenge in a review of the empirical literature (Petfield et al., 2015).

Practitioners identified parents as lacking insight in terms of outcomes of their behavior and in terms of how their children and others may feel and experience this behavior. This form of reflective capacity is similar to Fonagy’s model of metallization, impairments in which are common in individuals with BPD (Fonagy and Luyten, 2009). That metallization is further impaired by emotional arousal seems to find support within the accounts of parents and practitioners. However, within the interviews some, though not all parents, demonstrated a clear ability to reflect upon the effects their parenting may have had on their children. It is noteworthy that this was primarily the case for parents of older children who were reflecting back, rather than those who were in the more intense stage of active parenting, where arousal would be expected to be greatest. Reflection of this type was also exclusively associated with individuals who had participated in some form of psychological treatment.

Parents represented themselves as frequently lacking a familial and social support network. This related to the ability both to form and maintain relationships. This echoes studies that have found that individuals with BPD tend to have fewer social interactions, tend to describe social interactions more negatively, and are more likely to characterize their family and social network as “very poor” compared to groups with other disorders (Stepp et al., 2009; Beeney et al., 2018). Social network analysis of individuals with BPD identified a trend to “cut off” more people from their network than non-BPD controls. Individuals with BPD were also found to be less discriminant in their selection of individuals for social support (Clifton et al., 2007). Failure to select appropriate targets for support and closeness may lead to disappointment and rejection (for review on social interaction see Lis and Bohus, 2013). As one mother described “people came and went.” Cognitive mechanisms that have been implicated in these interpersonal difficulties include negative biases, impairment in interpretation of social behavior and social problem solving (for a review see and Lazarus et al., 2014). Whatever the cognitive mechanism, the functional impact of struggling to maintain friendships or close and supportive family relationships is the loss of practical and emotional support, including invaluable opportunities to share experiences of parenthood. Within the parent data, this deficit was mirrored by the clearly expressed desire for opportunities to connect with and learn alongside people with a shared understanding. Within the practitioner data it was reflected in the belief that parents needed opportunities to normalize experience, to “check in with other parents” (Clinical Psychologist).

### Limitations

While this study revealed a powerful picture of parenting experience, it was limited in terms of the number of parents involved and in the low number of male participants. Exploring the experience of male parents with BPD traits would be of value given the broad lack of research in male parents and some evidence of differential experiences of parental help seeking in males (Reupert and Maybery, 2009).

While every effort was made to capture a broad multi-disciplinary sample, the lack of representation from Child and Adolescent Mental Health Services and from Child Protection Services obscures part of the picture of the practice and understanding around these families. This is particularly relevant given the high incidence of child protection proceedings for parents with BPD traits (Adshead, 2015).

### Clinical Implications

Despite the clear need, parents with BPD traits describe limited, if any, access to targeted family-focused support. The experience of the interviewees in this study are borne out in the wider research literature (Stepp et al., 2012; Florange and Herpertz, 2019). In Australia, the multi-agency Project Air group has developed a modular package of parenting support designed to be incorporated into routine clinical practice. This has been found to have good clinical acceptability and ongoing use 12 months post the initial roll-out (McCarthy et al., 2016; Gray et al., 2019). In the United Kingdom, equivalent interventions are not available and parents (if they receive any service) are caught between standardized community interventions and BPD-focused treatment pathways. This is despite clear societal and economic benefits to supporting parents (Adshead, 2015).

In conducting this study we sought to illuminate the experience of parents with BPD traits and to determine the extent to which their experience was understood by the practitioners and helpers who work with them. What stood out to us above all was the clear love and good intentions all parents had toward their children. That these parents frequently lack decent parenting models to draw upon, coupled with the emotional and relational struggles they endure daily, make it essential that targeted parenting support is provided to these parents.

There is a clear clinical and preventative need for support which targets parents earlier and which is shaped by and sensitive to the impaired relational capacity of people who have not had positive formative relationship experience. Supporting parents and families earlier, i.e., intervening before the red flag of risk has been raised, has the potential to increase the acceptability of support and generate protective benefits for children. To enable this, practitioners need an empathic, non-stigmatizing narrative with which to talk to individuals with BPD traits about any mismatch between the parenting they offer and their child’s needs.

The parents interviewed expressed a clear need to feel understood and for opportunities to share experiences and utilize social learning. Practitioners and helpers need to be willing to get alongside these parents, to develop understanding of their situation and needs, and develop individualized and group-based programmes which reflect their findings and build upon what has been identified in this study. In this way they can appropriately support parents with strategies to develop a model of “good enough” parenting and to strip away some of the keenly felt stigma associated with being a parent with a complex and enduring mental illness.

## Data Availability Statement

Supporting data can be accessed via the ReShare repository of the UK Data Service (10.5255/UKDASN-854245).

## Ethics Statement

The studies involving human participants were reviewed and approved by the ethical approval was obtained from the NRES Committee Brighton and Hove. The patients/participants provided their written informed consent to participate in this study.

## Author Contributions

AD designed the study, analyzed the data, and wrote the manuscript. SC-H and HS designed the study, contributed to the interpretation of the data, and critically revised the manuscript. AP contributed to the analysis of the data and critically revised the manuscript. All authors contributed to the article and approved the submitted version.

## Conflict of Interest

The authors declare that the research was conducted in the absence of any commercial or financial relationships that could be construed as a potential conflict of interest.
