# Supplementary material for: The Parenting Experience of Those With Borderline Personality Disorder Traits: Practitioner and Parent Perspectives
Source: Front Psychol. 2020 Aug 7;11:1913. doi: 10.3389/fpsyg.2020.01913 (PMC7426472; doi:10.3389/fpsyg.2020.01913)
Supplement: Supplementary file 1 [file Data_Sheet_1.docx]

**Parent Topic Guide**

**The parenting experiences, help seeking and support provided to parents who struggle with emotional intensity and changeable moods (EICM)**

**Objectives:**

- To gain an understanding of parenting experiences of parents with emotional intensity struggles and changeable moods
- To gain an understanding of the core parenting struggles when parenting in the context of emotional intensity struggles and changeable moods.
- To gain an understanding of the aspects of parenting that those with EICM would like support with
- To identify strengths and weaknesses of any parenting support undertaken
- To identify targets for support and requirements of support

**Parenting experience:**

This section is focused on the parenting experience of participants and how it relates to their mental health problems

- Positives in relationship with child/being a parent/their child
- Challenges in relationship with child/being a parent
- Ways of being which get in the way of parenting
- Logistics – appointments etc
- Communicating with child and co-parent
- What changes in relationship and/or parenting

**Help seeking:**

This section focuses on the drivers for seeking support in parenting, what was offered and whether it was taken up

- When and why and what would be a point to seek help?
- Crisis
- Own / child behaviour
- Barriers to help seeking
- Barriers to participation in support offered

**Help received:**

For parents who participated in some form of support programme for parenting, this section is focused on understanding what worked, what didn’t and why

- Types of support
- Challenges
- Positives
- Interventions
  - Facilitation
  - Materials
  - Strengths / weaknesses
- Adherence

**Design:**

This section is focused on the elaboration of what parents would like to experience in parenting support

- Aims
- Structure
- Feeling safe
- Managing difficulties
- Contact
- Characteristics of the facilitators considered important
- Size
- Measurement of success

**Example questions**

- How would you describe your experience of parenting?
- It sounds like parenting was challenging, please can you tell me a little more about that?
- What would be helpful to you and your family in terms of supporting you with the challenges you have described?

**NHS Mental Health Services Practitioner Topic Guide:**

**The parenting experiences, help seeking and support provided to parents who struggle with emotional intensity and changeable moods (EICM)**

Objectives:

- To explore the way these practitioners conceptualise individuals struggling with EICM?
- To understand the extent to which practitioners engage with the parenthood and parenting of these individuals
- To explore the ways practitioners characterise working with parents struggling with EICM?
- To find out what parenting support parents are offered - strengths and weaknesses of existing support
- To explore mechanisms and opportunities to support these parents?

**Parenting while struggling with EICM:**

This section is focused on gaining an understanding of what these practitioners understand about these parents and the strengths and deficits in their parenting.

- Description in practitioners’ own language
- Diagnosis
  - To what extent does it feature in approach, decision making
- Traits and behaviours associated with these parents
- Parenting behaviours specific to or strongly associated with this group
- Crisis and risk
- Strengths and opportunities – targets for support

**Working with and supporting these parents:**

This section relates to the experience practitioners have working with parents who struggle with EUIM. Primarily this is focused on working with regard to their parenting but it can be opened up.

- Challenges and opportunities
  - Engagement
  - Emotional
  - Logistical
- Communication around parenting
  - Language
  - Fear of stigma
  - Triggering
- Managing parent’s fear/anxieties
- Strategies employed by practitioners in supporting parents (this may include avoidance and other maladaptive strategies – encourage honesty) and managing their own responses

**Specific parenting support/initiatives:**

This section is focussed more granularly on the types of support provided to parents struggling with EUIM. Want to gauge when, how and what is offered, how parents respond to it, how suitable and successful practitioners have found different types of support to be.

- Has it been offered
- Help sought/referred into/compulsion
- Types of support
- Suitability – practitioner/parent view
- What could be different/better

**Example Questions (from transcripts)**

- How would you describe in your own language the experience of individuals with BPD traits?
- How would you characterise or describe the parenting of individuals with BPD traits?
- Can you identify aspects of their parenting which are specific to these parents?

**Social Care Practitioner Topic Guide:**

**The parenting experiences, help seeking and support provided to parents who struggle with emotional intensity and changeable moods (EICM)**

**Objectives:**

- To explore the way these practitioners conceptualise individuals struggling with EICM?
- What strengths and challenges do practitioners they identify in their parenting
- To explore the ways practitioners characterise working with parents struggling with EICM?
- To find out and evaluate extant parenting support
- To explore practitioners’ views on possible targets and forms of support

**Parenting while struggling with EICM**

This section is focused on gaining an understanding of what these practitioners understand about the challenges and experiences of parents struggling with EICM as well as strengths and deficits in their parenting.

- Description in practitioners’ own language
- Diagnosis
  - To what extent does it feature in approach, decision making
- Traits and behaviours associated with these parents
- Parenting behaviours specific to or strongly associated with this group
- Crisis and risk
- Strengths and opportunities – targets for support

**Working with and supporting these parents**

This section relates to the experience practitioners have working with parents who struggle with EUIM. Primarily this is focused on working with regard to their parenting but it can be opened up.

- Challenges and opportunities
  - Engagement
  - Emotional fallout
  - Logistical
- Communication around parenting
  - Language
  - Fear of stigma
  - Triggering
- Managing parent’s fear/anxieties about Social Care
- Strategies employed by practitioners in supporting parents (this may include avoidance other maladaptive strategies – encourage honesty) and managing their own responses

**Specific parenting support/initiatives**

This section is focussed more granularly on the types of support provided to parents struggling with EICM. Want to gauge when, how and what is offered, how parents respond to it, how suitable and successful practitioners have found different types of support to be.

- When would it be offered
- Help sought/referred into/compulsion
- Types of support
- Suitability – practitioner/parent view
- What could be different/better

**Example Questions (from transcripts)**

- How would you describe in your own language the experience of individuals with BPD traits?
- How would you characterise or describe the parenting of individuals with BPD traits?
- Can you identify aspects of their parenting which are specific to these parents?

**Parenting Practitioner Topic Guide:**

**The parenting experiences, help seeking and support provided to parents who struggle with emotional intensity and changeable moods (EICM)**

**Objectives:**

- How do these practitioners conceptualise the parents?
- What strengths and challenges do practitioners they identify in their parenting
- How do practitioners find working with these parents?
- What parenting support are these parents offered – strengths and weaknesses of existing support
- What would the practitioners want to see developed to support these parents?

**Parenting while struggling with EICM**

This section is focused on gaining an understanding of what these practitioners understand about the challenges and experiences of parents struggling with EICM as well as strengths and deficits in their parenting.

- Description in practitioners’ own language
- Diagnosis
  - To what extent does it feature in knowledge, approach, decision making
- Traits and behaviours associated with these parents
- Parenting behaviours specific to or strongly associated with this group
- Crisis and risk
- Strengths and opportunities – targets for support

**Working with and supporting these parents**

This section relates to the experience practitioners have working with parents who struggle with EUIM. Primarily this is focused on working with regard to their parenting but it can be opened up.

- Challenges and opportunities
  - Engagement
  - Emotional fallout
  - Logistical
- Communication around parenting
  - Language
  - Fear of stigma
  - Triggering
- Managing parent’s fear/anxieties about Social Care
- Strategies employed by practitioners in supporting parents (this may include avoidance other maladaptive strategies – encourage honesty) and managing their own responses

**Specific parenting support/initiatives**

This section is focussed more granularly on the types of support provided to parents struggling with EUIM. Want to gauge when, how and what is offered, how parents respond to it, how suitable and successful practitioners have found different types of support to be.

- When would it be offered
- Help sought/referred into/compulsion
- Types of support
- Suitability – practitioner/parent view
- What could be different/better

**Example Questions (from transcripts)**

- How would you describe in your own language the experience of individuals with BPD traits?
- How would you characterise or describe the parenting of individuals with BPD traits?
- Can you identify aspects of their parenting which are specific to these parents?
